# Preventive Effects of Fucoidan and Fucoxanthin on Hyperuricemic Rats Induced by Potassium Oxonate

**DOI:** 10.3390/md17060343

**Published:** 2019-06-10

**Authors:** Yung-Tsung Chau, Hsin-Yuan Chen, Po-Han Lin, Shih-Min Hsia

**Affiliations:** 1 School of Nutrition and Health Sciences, College of Nutrition, Taipei Medical University, Taipei 11031, Taiwan; h7908262005@gmail.com (Y.-T.C.); hsin246@gmail.com (H.-Y.C.); phlin@tmu.edu.tw (P.-H.L.); 2 Graduate Institute of Metabolism and Obesity Sciences, College of Nutrition, Taipei Medical University, Taipei 11031, Taiwan; 3 School of Food and Safety, Taipei Medical University, Taipei 11031, Taiwan; 4 Nutrition Research Center, Taipei Medical University Hospital, Taipei 11031, Taiwan

**Keywords:** hyperuricemia, fucoidan, fucoxanthin, uric acid, xanthine oxidase

## Abstract

The purpose of this study was to investigate the preventive effects of fucoidan (Fc) and fucoxanthin (Fx) on hyperuricemic rats. Sprague Dawley (SD) rats were randomly assigned to seven groups: a control group, a hyperuricemia (HUA) group, low- and high-dose Fx groups, a Fc group, a combination Fc and Fx group, and a positive control group. Three weeks after the interventions, each group was given potassium oxonate (PO) and hypoxanthine (HX) to induce HUA in all groups except for the control group, and the rats were then sacrificed. Blood and urine were analyzed for biochemical properties, and differences in urine volume were determined. Livers and kidneys were collected to analyze xanthine oxidase (XO) activity and the expression of uric acid (UA) transporter-related proteins (GLUT9, ABCG2, OAT1, URAT1). The results show that HUA was successfully induced by PO/HX after 4 h of administration. The activity of XO was significantly reduced by a combination of Fc and Fx. In the combination group, both ABCG2 and OAT1 increased significantly, whereas GLUT9 and URAT1 decreased significantly. In summary, the combination of Fc and Fx can inhibit the activity of XO in the liver and regulate the expression of proteins related to UA transporter in the kidney to reduce the UA level in serum.

## 1. Introduction

Hyperglycemia, hyperlipidemia, and hypertension are currently the top three major chronic diseases in Taiwan. According to the Nutrition and Health Survey in Taiwan (NAHSIT) data from 2005 to 2008, the prevalence of hyperuricemia (HUA) in Taiwan has increased each year. Further, hyperuricemia has gradually become the fourth most prevalent chronic disease [1]. High uric acid (UA) concentrations in the body that persist for a long time can increase the risk of gout, cardiovascular disease, kidney-related diseases, diabetes, hypertension, and hyperlipidemia. The current treatment of hyperuricemia is based on pharmaceutical intervention, which is supplemented by changes in diet and lifestyle. However, long-term use of medication to lower uric acid levels may cause severe allergic reactions, kidney injury, and even death [2].

In the kidney, there are millions of renal tubular cells with different uric acid-related transporter proteins on either their apical or basolateral membrane [3]. Different proteins are responsible for regulating the concentration of uric acid in the body. Urate transporter protein 1 (UTAT1), a protein on the apical membrane in the proximal tubular epithelial cells, is responsible for regulating the reabsorption of uric acid into renal proximal tubule epithelial cells. Glucose transporter protein 9 (GLUT9) is located on the outside of the basolateral membrane of the renal tubules, and it regulates the reabsorption of urate into the blood to control the amount of uric acid that enters the bloodstream. The organic anion transporter (OAT1) is present on the basolateral membrane of the renal tubule, and its major function is to uptake urate anions from the circulatory system and secrete them into tubular cells. The human ATP-binding cassette (subfamily G2, ABCG2) is a protein that exists on the apical membrane of the proximal convoluted tubule of the kidney, and it is responsible for regulating urate and secreting it into the collecting duct for excretion.

Many recent studies have investigated the effects of ingesting natural extracts to prevent or ameliorate hyperuricemia. Fucoidan (Fc) is a sulfated fucose-rich polysaccharide that is present in high levels in brown seaweed and has been shown to have anticancer and antioxidant effects in animal experiments [4]. Fucoxanthin (Fx) is a red-orange carotenoid that is extracted from natural seaweed. It has been reported to have a potential in improving obesity [5] and having anti-cancer effects [6]. Moreover, the combination of Fc and Fx treatment has been reported to have a role in improving the glucose homeostasis and lipid metabolism in the type II diabetes mouse model [7], as well as in improving the ventricular rhythm and muscular function of the aging mouse model [8]. However, the effect of combining Fc and Fx to prevent hyperuricemia is still unclear. Therefore, the purpose of this study was to investigate whether fucoidan and fucoxanthin have a preventive effect on hyperuricemia.

## 2. Results

### 2.1. Uric Acid Level at Different Time Points in Potassium Oxonate and Hypoxanthine (PO/HX)-Induced Acute Hyperuricemic Rats

**Figure 1:**
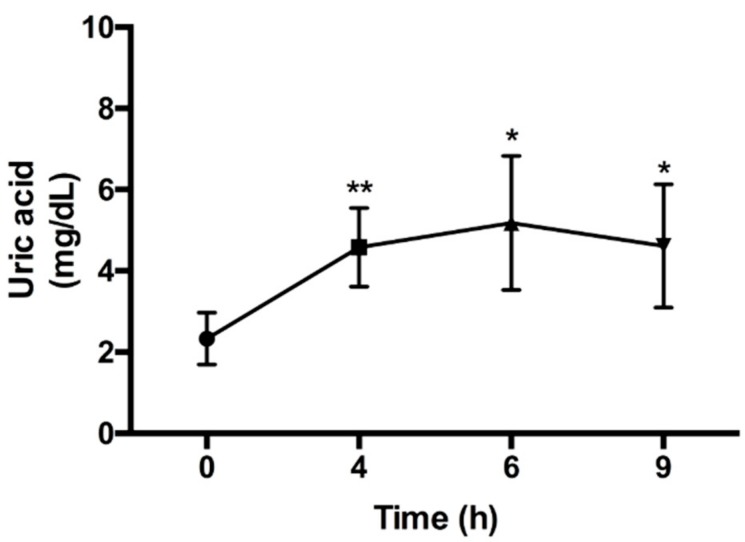
Uric acid (UA) levels at different time points in potassium oxonate and hypoxanthine (PO/HX)-induced acute hyperuricemic rats. Blood samples were collected before inducing the hyperuricemia model (PO: 300 mg/kg; HX: 300 mg/kg) at the first time point (0 h) as the baseline. At the 4th, 6th, and 9th time points after the hyperuricemia model was induced, blood samples were collected from the tail vein at different time intervals. Values are presented as the mean ± SEM (*n* = 9) (* *p* < 0.05, ** *p* < 0.01) and were determined by paired *t*-test.

A hyperuricemic rat model was generated by oral gavage of potassium oxonate (PO, 300 mg/kg) and hypoxanthine (HX, 300 mg/kg). At 0, 4, 6, and 9 h after the induction of acute hyperuricemic rats, blood was collected from the tail vein, and the serum uric acid concentration was determined. The results showed that the uric acid level was increased at 4, 6, and 9 h after induction. It reached a peak at the 6th hour (Figure 1). Therefore, the induction period used for further experiments was the 4th hour after the induction. 

### 2.2. Effects of Fucoxanthin and Fucoidan on Body Weight in Hyperuricemic Rats

**Figure 2:**
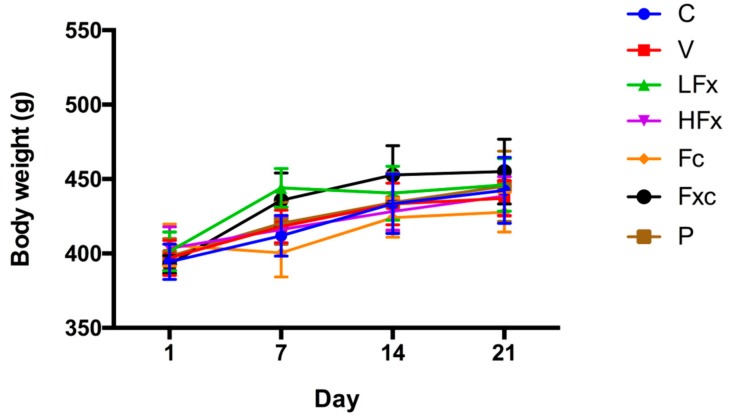
Preventive effects of fucoxanthin (Fx) and fucoidan (Fc) on body weight in hyperuricemic rats. Values are presented as the mean ± SEM (*n* = 3–5). #*p* < 0.05, ##*p* < 0.01 versus control group. *p < 0.05, **p < 0.01 versus vehicle group. C, control group; V, vehicle group (PO: 300 mg/kg + HX: 300 mg/kg); LFx, low-dose fucoxanthin group (Fx: 150 mg/kg); HFx, high-dose fucoxanthin group (Fx: 300 mg/kg); Fc, fucoidan group (Fc: 150 mg/kg); Fxc, low-dose fucoxanthin (Fx: 150 mg/kg) + fucoidan group (Fc: 150 mg/kg); P, positive control group (ALL: 20 mg/kg).

Hyperuricemia was induced three weeks after the onset of the fucoxanthin and fucoidan interventions. The results showed that there was no significant difference in body weight between groups for the same time periods, as shown in Figure 2.

### 2.3. Effects of Fucoxanthin and Fucoidan on Tissue Weight in Hyperuricemic Rats

Acute hyperuricemia was induced three weeks after the onset of the fucoxanthin and fucoidan interventions. At the end of the experiment, the rats were sacrificed and then tissue weights were monitored. The results showed that there were no significant differences in the weight of the heart, liver, kidneys, spleen, or testes between the groups. The results are shown in Table 1.

### 2.4. Treatment of Hyperuricemic Rats with Fucoxanthin and Fucoidan Improved Serum Uric Acid Level

**Figure 3:**
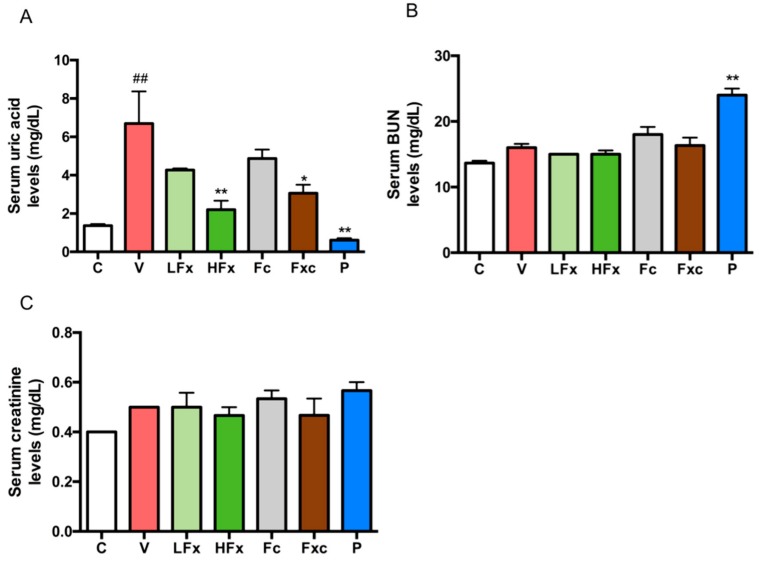
Effects of fucoxanthin and fucoidan on (**A**) serum UA, (**B**) serum blood urea nitrogen (BUN), and (**C**) serum creatinine in hyperuricemic rats. Values are presented as the mean ± SEM (*n* = 3–5). #*p* < 0.05, ##*p* < 0.01 versus control group. *p < 0.05, **p < 0.01 versus vehicle group. C, control group; V, vehicle group (PO: 300 mg/kg + HX: 300 mg/kg); LFx, low-dose fucoxanthin group (Fx: 150 mg/kg); HFx, high-dose fucoxanthin group (Fx: 300 mg/kg); Fc, fucoidan group (Fc: 150 mg/kg); Fxc, low-dose fucoxanthin (Fx: 150 mg/kg) + fucoidan group (Fc: 150 mg/kg); P, positive control group (ALL: 20 mg/kg).

After three weeks of intervention with fucoxanthin and fucoidan, the hyperuricemia model was induced. The results showed that the serum uric acid level of the vehicle group was significantly higher than that of the control group, whereas the positive control group had a significantly lower uric acid level than the vehicle group. This finding demonstrates that the hyperuricemia model was successfully induced. In the interventional groups, both low-dose and high-dose fucoxanthin groups tended to have lower serum uric acid levels, and the serum uric acid level significantly reduced in the high-dose fucoxanthin group compared with the vehicle group. There was no significant difference in the uric acid levels of the fucoidan group compared with the vehicle group. However, the group treated with a combination of low doses of both fucoxanthin and fucoidan had significantly lower uric acid levels than the vehicle group (Figure 3A). Moreover, the serum levels of blood urea nitrogen (BUN) and creatinine were detected to evaluate the renal function. The results showed that there was no significant difference in blood urea nitrogen (BUN) between the groups, except for the positive control group compared with the vehicle group (Figure 3B). There was no significant difference in serum creatinine concentration between the groups (Figure 3C).

### 2.5. Effects of Fucoxanthin and Fucoidan on Urine Volume, Urine Uric Acid, Fractional Excretion of Uric Acid (FEUA), and Clearance Creatinine Rate in Hyperuricemic Rats

**Figure 4:**
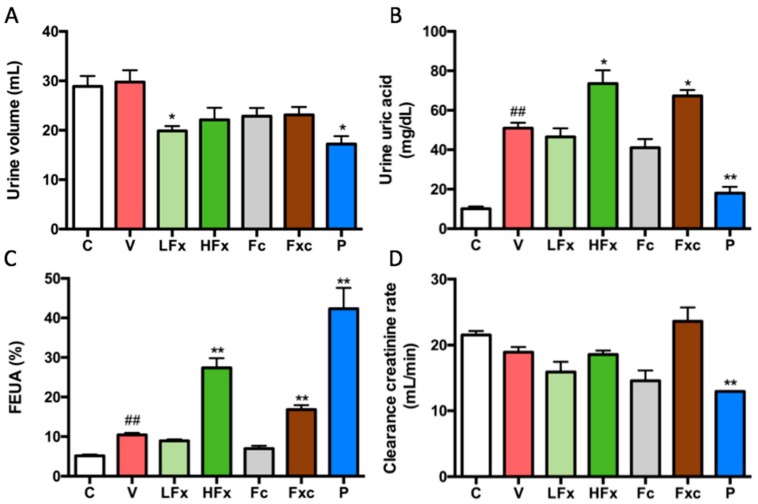
Effects of fucoxanthin and fucoidan on (**A**) urine volume, (**B**) urine uric acid, (**C**) fractional excretion of uric acid (FEUA), and (**D**) clearance creatinine rate in hyperuricemic rats. Values are presented as the mean ± SEM (*n* = 3). #*p* < 0.05, ##*p* < 0.01 versus control group. *p < 0.05, **p < 0.01 versus vehicle group. C, control group; V, vehicle group (PO: 300 mg/kg + HX: 300 mg/kg); LFx, low-dose fucoxanthin group (Fx: 150 mg/kg); HFx, high-dose fucoxanthin group (Fx: 300 mg/kg); Fc, fucoidan group (Fc: 150 mg/kg); Fxc, low-dose fucoxanthin (Fx: 150 mg/kg) + fucoidan group (Fc: 150 mg/kg); P, positive control group (ALL: 20 mg/kg).

The results showed that there was no significant difference in urine volume between the control group and the vehicle group. However, there was a difference in volume for each intervention group and the positive control group compared with the vehicle group, and it had a decreasing trend. The low-dose fucoxanthin group and the positive control group had a significantly lower urine volume compared with the vehicle group (Figure 4A). In the urine, the uric acid level of the vehicle group was significantly increased compared with the control group. In the intervention group, the high-dose fucoxanthin group and the combination of fucoidan and low-dose fucoxanthin had a significantly increased concentration of urine uric acid compared with the vehicle group, whereas the positive control group compared with the vehicle group had a significantly decreased concentration (Figure 4B). The fractional excretion of uric acid (FEUA) was significantly increased in the vehicle group compared with the control group. The increased FEUA in the high dose of fucoxanthin, the combination of fucoidan and low-dose fucoxanthin, and allopurinol as a positive control was observed, but there was no significant difference between the low-dose fucoxanthin and the fucoidan group compared with the vehicle group (Figure 4C). Finally, there was no significant difference in the creatinine clearance rate between the groups, except for the positive control group (Figure 4D).

### 2.6. Effects of Fucoxanthin and Fucoidan on Xanthine Oxidase (XO) Activity in the Liver of Hyperuricemic Rats

**Figure 5:**
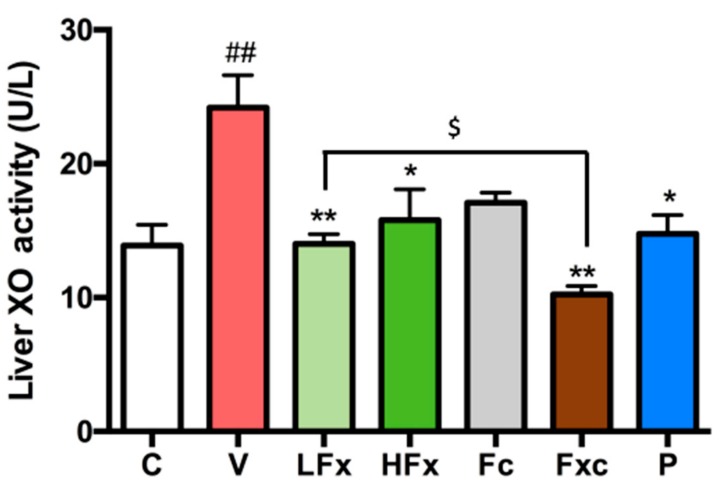
Effects of fucoxanthin and fucoidan on XO activity in liver of hyperuricemic rats. Values are presented as the mean ± SEM (*n* = 3). #*p* < 0.05, ##*p* < 0.01 versus control group. *p < 0.05, **p < 0.01 versus vehicle group. $*p* < 0.05 versus low-dose fucoxanthin group. C, control group; V, vehicle group (PO: 300 mg/kg + HX: 300 mg/kg); LFx, low-dose fucoxanthin group (Fx: 150 mg/kg); HFx, high-dose fucoxanthin group (Fx: 300 mg/kg); Fc, fucoidan group (Fc: 150 mg/kg); Fxc, low-dose fucoxanthin (Fx: 150 mg/kg) + fucoidan group (Fc: 150 mg/kg); P, positive control group (ALL: 20 mg/kg).

The results of this experiment showed that the activity of xanthine oxidase was significantly increased in the vehicle group compared with the control group. The groups receiving the low dose of fucoxanthin, the high dose of fucoxanthin, the combination of fucoidan and low-dose fucoxanthin, and allopurinol as a positive control had significantly reduced xanthine oxidase activity. However, the intervention group that received only fucoidan had no significant difference from the vehicle group. In addition, there was a significant difference between the group receiving a combination of the two extractions and the low-dose fucoxanthin group, as shown in Figure 5.

### 2.7. Effects of Fucoxanthin and Fucoidan on the Expression of Urate-Related Transporter Proteins in the Kidney of Hyperuricemic Rats

**Figure 6:**
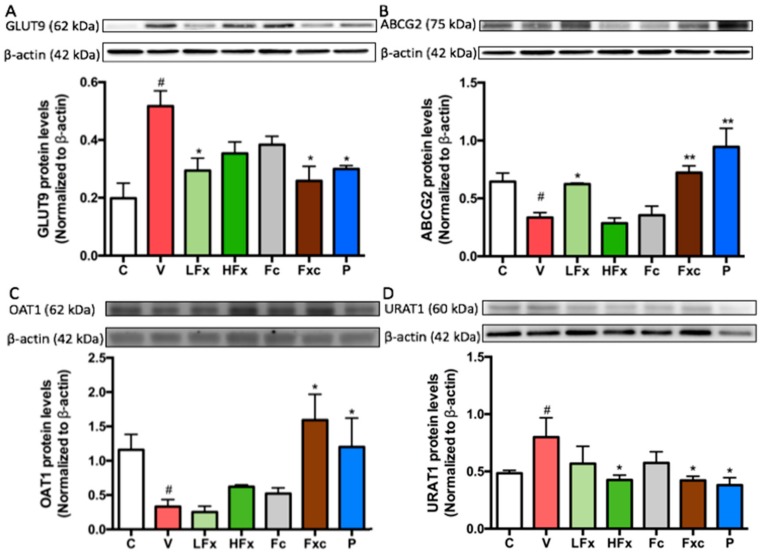
Effects of fucoxanthin and fucoidan on the expression of urate-related transporter proteins (**A**) GLUT9, (**B**) ABCG2, (C) OAT1 and (**D**) URAT1 in the kidneys of hyperuricemic rats. Values are presented as the mean ± SEM (*n* = 3). #*p* < 0.05, ##*p* < 0.01 versus control group. *p < 0.05, ** *p* < 0.01 versus vehicle group. $*p* < 0.05 versus low-dose fucoxanthin group. C, control group; V, vehicle group (PO: 300 mg/kg + HX: 300 mg/kg); LFx, low-dose fucoxanthin group (Fx: 150 mg/kg); HFx, high-dose fucoxanthin group (Fx: 300 mg/kg); Fc, fucoidan group (Fc: 150 mg/kg); Fxc, low-dose fucoxanthin (Fx: 150 mg/kg) + fucoidan group (Fc: 150 mg/kg); P, positive control group (ALL: 20 mg/kg).

According to the results, the expression of the uric acid-related transporter protein GLUT9 in rat kidneys was significantly increased in the vehicle group compared with the control group. After the PO/HX induction (after three weeks of intervention), the groups receiving a low dose of fucoxanthin and a combination of the two extractions had significantly decreased GLUT9 among the hyperuricemia groups. Compared with the vehicle group, the high-dose fucoxanthin group and the fucoidan group showed no significant difference in expression. Of course, GLUT9 expression in the positive control group significantly differed from that in the vehicle group (Figure 6A). On the other hand, the expression of ABCG2 was significantly decreased in the vehicle group compared with the control group. In addition, compared with the vehicle group, ABCG2 expression was significantly increased in the low-dose fucoxanthin group and the combination of fucoidan and low-dose fucoxanthin group compared with the vehicle group. There was no significant difference for the high-dose fucoxanthin group and the fucoidan group compared with the vehicle group. There was a significant increase in the expression of ABCG2 in the positive control group compared with the vehicle group (Figure 6B). OAT1 protein expression was significantly lower in the vehicle group than the control group. After the interventions, there was no significant difference in the expression of OAT1 between the vehicle group and the groups receiving a low dose of fucoxanthin, a high dose of fucoxanthin, and a low dose of fucoidan. However, the combination of fucoidan and low dose of fucoxanthin resulted in the significantly increased expression of OAT1 compared with the vehicle group (Figure 6C). Moreover, the vehicle group had a significantly increased expression of URAT1 compared with the control group. There was no significant difference between the group that was administered low doses of both fucoxanthin and fucoidan compared with the vehicle group. Compared with the vehicle group, the group receiving a high dose of fucoxanthin and the group receiving a combination of low doses of the two extractions both had significantly decreased URAT1 expression (Figure 6D).

## 3. Discussion

The hyperuricemia rodent model can be induced through different inhibition pathways as follows. (1) Enhancement of the uric acid level in the body by the administration of uric acid directly [9], high-purine food such as fructose [10], or uric acid precursors, such as hypoxanthine (Hx) [11], which can produce uric acid to establish the model. (2) Inhibition of the excretion of uric acid through the kidneys. The treatment of adenine or ethambutol [12] can prevent uric acid excretion from the kidneys, leading to an increase in the concentration of uric acid in the serum. (3) Inhibition of uricase activity, which is a common model in rodent experiments. Potassium oxonate (PO), an inhibitor of uricase, can be used to suppress uricase activity through competing with uric acid for binding to uricase [13,14,15]. (4) A hyperuricemia animal model can be obtained by knocking down the uricase gene in rodents [16]. However, for better induction results, some studies have combined two or three methods. In the present study, the treatment with uricase inhibitor potassium oxonate (PO, 300 mg/kg) alone was not satisfactory after 12 h. We found that the uric acid levels in each group were not different from those in the control group (data not shown). Therefore, we modified the strategy, and the treatment of rats with a combination of PO (300 mg/kg) and HX (HX, 300 mg/kg) at 4 h significantly increased the serum uric acid concentration. Therefore, this model was used to perform further experiments in this study. 

It has been reported that after the induction of rat hyperuricemia status by treatment with adenine (200 mg/kg) and ethambutol (250 mg/kg) for 10 days, the kidney-to-body weight ratio (kidney weight index) of the hyperuricemia group was significantly increased compared with the control group. The pale, swollen, and coarse morphology of the kidney was observed, which means that the kidney underwent a pathological change in the hyperuricemia [12]. Researchers have also mentioned that in their experiment on hyperuricemia induced by adenine (0.1 g/kg) with potassium oxonate (1.5 g/kg) for six weeks, the kidney weight index of the hyperuricemia group was significantly increased compared with the control group [17]. Moreover, rats were administered potassium oxonate (250 mg/kg) daily, along with a subcutaneous injection of hypoxanthine (10 mg/kg) for two weeks. The results showed a significant increase in the kidney weight index in the hyperuricemia group compared with the control group [18]. In the present study, the acute hyperuricemic rat model was generated by co-treatment with potassium oxonate (300 mg/kg) combined with hypoxanthine (300 mg/kg) for four hours. According to our result, the kidney index was not altered in the hyperuricemic rat group, suggesting that the induction time in our model was shorter than that in the previous study, and a significant difference between the hyperuricemia group and each of the other groups could not be observed.

The level of serum blood urea nitrogen (BUN) and creatinine was used to evaluate the kidney function. Previously, it has been reported that one hour after hyperuricemia was induced by potassium oxonate, interventions comprising different concentrations of astilbin (5, 10, 20 mg/kg) were applied for seven consecutive days. The results showed that the BUN and creatinine in the serum level in the hyperuricemia group were significantly increased compared with those in the control group. After the intervention, both BUN and creatinine significantly decreased in a dose-dependent manner [19]. In addition, the plasma BUN and serum creatinine levels were increased in the adenine/ethambutol-induced hyperuricemic rat, and this symptom can be rescued by intervention with taurine that was applied for seven days [12]. In our study, both serum BUN and creatinine were not altered, suggesting that the acute PO/HX-induced hyperuricemic rat model in this study did not cause any kidney damage. However, the BUN level in the allopurinol treatment (as a positive control) group was significantly increased compared with the hyperuricemia group, implying that the kidney function may become impaired by long-term administration of allopurinol. In agreement with our results, patients with allopurinol hypersensitivity has been reported to have a higher risk for renal or cardiovascular disease.

It has been shown that the uric acid level in the urine was increased by seven days of oral gavage with potassium oxonate (250 mg/kg) in rats; however, this status could be rescued by administration with polydatin (25, 50 mg/kg) [3]. In addition, treatment with liquiritigenin (20 and 40 mg/kg) has been reported to have a potential role in improving the higher urine uric acid level in PO (250 mg/kg)-induced hyperuricemic rats [14]. In our study, the concentration of uric acid in the urine was increased in the hyperuricemia group. However, the higher urine uric acid level was observed in the high-dose fucoxanthin group and the combination group. The high-dose fucoxanthin group and the combination group also showed a significant increase in the fractional excretion of uric acid (FEUA) compared with the hyperuricemia group, suggesting that a high-dose of fucoxanthin alone or a combination of fucoidan and fucoxanthin treatment could promote the uric acid excretion from the body to the urine. It has been shown that the PO (250 mg/kg)-induced hyperuricemia group had a lower creatinine clearance than the control group. After treatment with liquiritigenin (20, 40 mg/kg), the creatinine clearance was increased compared with that in the hyperuricemia group [14]. In the present study, the creatinine clearance in the hyperuricemia group was not altered. There was no significant difference between the hyperuricemia group and any intervention group, except for the positive control group. These results cause us to speculate that the difference between the hyperuricemia group and the positive control group is due to the side effects of allopurinol; however, the acute induction model was not long enough to cause renal damage.

A previous study has reported that the hepatic xanthine oxidase activity did not cause any alteration in the PO (250 mg/kg)- and HX (300 mg/kg)-induced hyperuricemic rat model [11]. In addition, there was no significant difference in the positive control group, which was given a dose of 3.5 mg/kg allopurinol, compared with the hyperuricemia group [11]. However, according to our results, the PO (300 mg/kg)- and HX (300 mg/kg)-induced hyperuricemic rats had a significantly increased xanthine oxidase activity in the liver, and this stimulatory effect was rescued by intervention with fucoxanthin alone or combined with fucoxanthin and fucoidan. Moreover, the combination group had the best efficiency of xanthine oxidase inhibition. 

**Figure 7:**
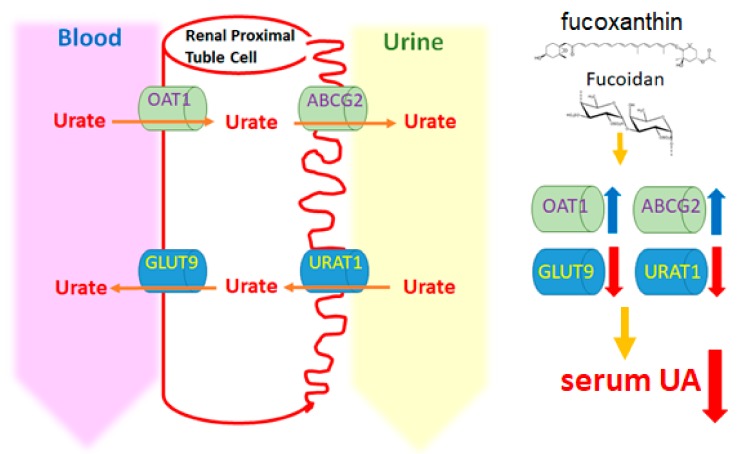
Proposed mechanism of fucoidan- and fucoxanthin-mediated modulation of the uric acid metabolism pathway in hyperuricemic rats. The diagram demonstrates that fucoidan and fucoxanthin may inhibit hyperuricemia.

Previous studies have shown that the expression levels of the two transporters, GLUT9 and URAT1, in the kidney were significantly increased in the PO (300 mg/kg)-induced hyperuricemia group, but the higher GLUT9 and URAT1 protein levels in the hyperuricemia group were reduced by intervention with luteolin (20, 40, 100 mg/kg) [20]. In the hyperuricemia mouse model, the hyperuricemia-induced high renal GLUT9 protein expression was reduced by treatment with nuciferin. In addition, the expression of the ABCG2 and OAT1 transporter proteins in the hyperuricemia group was significantly lower than that of the control group, whereas treatment of hyperuricemia mice with nuciferin significantly increased the expression of ABCG2 and OAT1 proteins [21]. In the present study, the results showed that the expression levels of GLUT9 and URAT1 were significantly increased in the hyperuricemia group, whereas the expression levels of ABCG2 and OAT1 were significantly decreased. Among the intervention groups, those receiving fucoxanthin alone and a combination of fucoxanthin and fucoidan had a significantly reduced expression level of GLUT9 and URAT1 proteins. The protein expression of ABCG2 was significantly increased in the low-dose fucoxanthin group and the combination group compared with the hyperuricemia group. Finally, the protein expression of OAT1 was only significantly increased in the combination group compared with the hyperuricemia group. These results suggested that only the combination group (fucoidan and low dose fucoxanthin) significantly regulated all uric acid-related transporters in the kidney compared with the hyperuricemia group. The mechanism by which fucoxanthin and fucoidan modulate hyperuricemia is proposed in Figure 7.

It has been reported that hyperuricemia patients with a higher level of uric acid in the serum are exposed to a higher risk of cardiovascular disease [22]. Uric acid has been reported to inhibit nitric oxide (NO) production and induce inflammation cytokine interleukin (IL)-6, IL-8, and tumor necrosis factor (TNF)-α release from human umbilical vein endothelial cells, which are associated with endothelium injury and vascular dysfunction [23]. In addition, uric acid can pass through the blood–brain barrier and induce hypothalamic inflammation through activation of the NFκB-mediated inflammation pathway [24]. However, fucoidan and fucoxanthin have been reported to have a potential role in improving inflammation via suppressing inflammatory cytokine production [25]. Treatment with fucoidan has also been reported to improve renal tubulointerstitial fibrosis in a chronic kidney disease mouse model via reducing the β-catenin and TGF-β1 expression [26]. According to our findings in this study, we propose that a combined treatment with fucoidan and fucoxanthin reducing the uric acid level may improve the inflammatory signaling in the hyperuricemic rat.

## 4. Materials and Methods

### 4.1. Materials

Hi-Q oligo-fucoidans (Fc) and high-stability fucoxanthin (HS Fucoxanthin, HSFUCO) (Fx) were derived from *Laminaria japonica* and obtained from Hi-Q Marine Biotech International Ltd. (New Taipei City, Taiwan) [27]. Oxonic acid (potassium salt), hypoxanthine, and the xanthine oxidase fluorometric assay kit were purchased from Cayman Chemical (Ann Arbor, MI, USA). Allopurinol was purchased from Sigma-Aldrich (St. Louis, MO, USA). Rabbit anti-ABCG2 and anti-OAT1 antibodies were obtained from Abcam (Cambridge, MA, USA). Rabbit anti-GLUT9 antibody was obtained from Millipore (Billerica, MA, USA), and rabbit anti-URAT1 antibody was obtained from Proteintech (Rosemont, IL, USA).

### 4.2. Animals

For this study, 35 male Sprague Dawley (SD) rats (9 to 10 weeks old, 400 ± 20 g) were purchased from BioLASCO Taiwan Co., Ltd. (Yilan, Taiwan). The experimental animals were quarantined and allowed to acclimate for a week before the experiment. The room temperature was maintained at 21 ± 2 °C, and the relative humidity was kept at 40–60%. Two or three animals were housed per cage under standard laboratory conditions, with a 12/12 h light/dark cycle. During the whole experimental period, food and water were provided ad libitum. All the experiments involving animals were approved by the Institutional Animal Care and Use Committee (IACUC), with approval number LAC-2018-0085 (Approval day: 12th June 2018), Taipei Medical University, Taiwan. The experiment complied with the Guide for the Care and Use of Laboratory Animals published by the National Research Council (revised 2011) and the Guide for the Care and Use of Laboratory Animals—Taiwanese Edition (1996). 

### 4.3. Hyperuricemic (HUA) Rats and Drug Administration

Potassium oxonate (PO), an uricase inhibitor, was used to induce hyperuricemia. Hypoxanthine (HX) is a precursor of uric acid, and allopurinol, a xanthine oxidase inhibitor, is a clinical medication for hyperuricemia. In this study, PO (300 mg/kg) was mixed with 0.5% (w/v) sodium carboxymethyl cellulose solution (CMC, Sigma-Aldrich, St Louis, MO, USA) and HX (300 mg/kg) were dissolved in distilled water. First, nine rats were used to induce the acute hyperuricemia (HUA) rat model by oral gavage of PO and HX. Blood samples were collected through the tail veil in a time-course manner, and then serum uric acid was measured. According to this experiment, treatment of PO/HX for 4 h was selected to design the next experiment. 

After a one-week recovery, these nine rats and the other twenty-six rats (total of 35 rats) were randomly divided into seven groups (*n* = 5 per group): (1) control group (C) rats were treated with 0.5% CMC; (2) vehicle group (V)rats were treated with 0.5% CMC; (3) low-dose fucoxanthin group (LFx) rats were treated with 150 mg/kg of fucoxanthin; (4) high-dose fucoxanthin group (HFx) rats were treated with 300 mg/kg of fucoxanthin; (5) fucoidan group (Fc) rats were treated with 150 mg/kg of fucoidan; (6) combination of low-dose fucoxanthin and fucoidan group (Fxc) rats were co-treated with 150 mg/kg of fucoxanthin and 150 mg/kg of fucoidan; and (7) positive control group (P) rats were treated with 20 mg/kg of allopurinol. These interventions were performed by oral gavage once daily for 21 days. 

**Figure 8:**
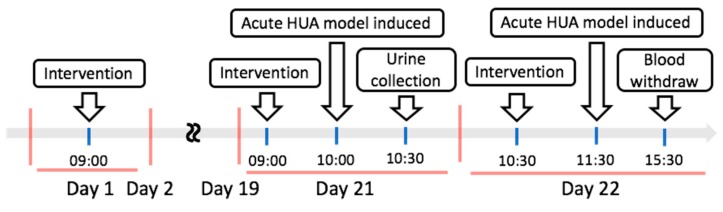
Schematic design of the in vivo hyperuricemic rat model.

One hour after the intervention on the 21st day, the hyperuricemia (HUA) model was induced by oral gavage of PO and HX, and then the rats were housed in metabolic cages and the urine was collected for 24 h. The urine sample was centrifuged (3000× *g*, 4 °C) for 10 min to obtain urine free of particulate contaminants. The volume of urine was measured using a graduated cylinder. On the next day, a process was performed similar to the one followed above on the 21st day; the hyperuricemia model was induced again (Figure 8). After 4 h, all animals were sacrificed using a Zoletil (20 mg/kg) + rompun (5 mg/kg) mixed solution (*v*/*v* = 1:1) anesthesia. 

### 4.4. Blood, Urine, and Tissue Sample Collection

After the rats were sacrificed, blood samples were collected by cardiac puncture and allowed to clot for at least 1 h at room temperature and then centrifuged (3000× *g*, 4 °C) for 10 min, after which the serum was collected. On the day of collection, the serum and urine samples from the metabolic cage collection were delivered to Taipei Medical University Hospital (Taipei, Taiwan) for biochemical examination. Kidneys, livers, hearts, spleens, and testes were quickly excised, weighed, and immediately stored at −80 °C for further analysis.

### 4.5. Determination of Uric Acid, BUN, and Creatinine Levels in Serum

Twenty-four hours before the sacrifice, all animals were placed in metabolic cages to collect fasting urine. After the urine volume was measured using a graduated cylinder (Sigma-Aldrich, St. Louis, MO, USA), the serum and urine samples were delivered to the Taipei University of Medical Sciences. The uric acid concentration, blood urea nitrogen, and creatinine in the serum and urine were determined by using a chemical analyzer (Roche Diagnostics, Rotkreuz, Switzerland). After the biochemical data were collected, the fractional excretion of uric acid (FEUA) and creatinine clearance rate (CCr) were calculated using the following equations:

FEUA (%) = (UUA × SCr)/(SUA × UCr) × 100,

CCr (mL/min) = (UCr × Urine volume 24h)/(SCr × 1440 min).

### 4.6. Xanthine Oxidase Assay

After the livers were homogenized and centrifuged, the supernatant was collected. The xanthine oxidase activities were detected using a xanthine oxidase fluorometric assay kit (Cayman Chemical, Ann Arbor, MI, USA). The procedures were followed according to the manufacturer’s instructions.

### 4.7. Western Blot Analysis

Tissue protein levels were analyzed by Western blot as described previously [27,28]. After kidney samples were homogenized at 4 °C, they were separated by protein electrophoresis and Western blot to analyze the expression of kidney-related transporter proteins. Lysis buffer (RIPA/protein inhibitor = 19:1) was added to the kidney homogenization and further homogenized by an organization homogenizer (Qiagen, Chatsworth, CA, USA) (1/20 Hz, 30 s/time). After centrifuging the sample for 30 min at 12,000 rpm and 4 °C, the supernatant was collected, and the protein sample was quantified using a BCA (bicinchoninic acid) assay. A volume equivalent to 80 μg of the sample protein was taken and mixed with 1/5 of the total volume of sample dye (4×). The mixture was left to react at 100 °C for 5 min to facilitate protein denaturation. Then, the protein extraction was added to a 10% SDS gel for protein electrophoresis. When the proteins were adequately separated, the protein was transferred to 0.45 μm PVDF membrane, which was then blocked with blocking buffer for 1 h and washed with TBST three times (10 min/wash). After washing the membrane, the membrane was incubated with primary antibodies at 4 °C overnight; then, the membrane was washed with TBST three times (10 min/wash). Subsequently, the membranes were incubated with anti-rabbit or anti-mouse IgG. The uric acid-related transporter proteins were detected by a chemiluminescence detection system according to the manufacturer’s instructions (ECL, T-Pro Biotechnology, New Taipei City, Taiwan).

### 4.8. Statistical Analysis

All data were expressed as the mean ± standard error of the mean (SEM). The effect of PO/HX on the induction of the hyperuricemia model was analyzed by paired *t*-test. The effects of fucoidan and fucoxanthin on biochemical composition and renal function were analyzed by one-way analysis of variance (ANOVA) and unpaired *t*-test to determine the level of significance. The results of Western blotting were analyzed using the Mann–Whitney test. A value of *p* < 0.05 was considered statistically significant. Figures were obtained using the Statistical Analysis System (GraphPad Prism 6, GraphPad Software, Inc., San Diego, CA, USA).

## 5. Conclusions

According to current research, this study is the first to investigate the effects of administering fucoidan, fucoxanthin, and a combination of the two on hyperuricemia. The results of this study showed that the administration of fucoidan (150 mg/kg) and fucoxanthin (150 mg/kg) significantly inhibited the activity of xanthine oxidase in the liver. The protein expression of ABCG2 and OAT1 in the kidney was increased, and the protein expression of GLUT9 and URAT1 was downregulated; these effects reduce serum uric acid concentration, thereby preventing the occurrence of hyperuricemia. This study found that the combination of fucoidan and fucoxanthin can significantly inhibit the activity of xanthine oxidase and regulate the expression of uric acid-related transporters. Further study directions include focusing on the genetic level of regulation or other pathways of this phenomenon. In addition, this study established the preventive effect of these two interventions on hyperuricemia.

## Figures and Tables

**Table 1:** Effects of fucoxanthin and fucoidan on tissue weights in PO/HX-induced hyperuricemic rats. All values are normalized with body weight. The values are presented as the mean ± SEM (*n* = 3–5) and determined by one-way ANOVA.

Tissue (g)	C	V	LFx	HFx	Fc	Fxc	P
Kidneys	0.73 ± 0.02	0.80 ± 0.03	0.83 ± 0.04	0.77 ± 0.04	0.91 ± 0.07	0.81 ± 0.04	0.91 ± 0.08
Liver	2.96 ± 0.08	2.82 ± 0.08	2.82 ± 0.10	2.37 ± 0.46	2.66 ± 0.06	2.80 ± 0.12	2.74 ± 0.04
Heart	0.30 ± 0.01	0.30 ± 0.01	0.29 ± 0.01	0.31 ± 0.02	0.28 ± 0.01	0.29 ± 0.01	0.31 ± 0.01
Spleen	0.17 ± 0.01	0.16 ± 0.00	0.16 ± 0.01	0.15 ± 0.01	0.16 ± 0.01	0.21 ± 0.05	0.17 ± 0.01
Testes	0.76 ± 0.04	0.80 ± 0.03	0.75 ± 0.04	0.73 ± 0.07	0.80 ± 0.04	0.79 ± 0.04	0.77 ± 0.05
